# Reverse Engineering: A Key Component of Systems Biology to Unravel Global Abiotic Stress Cross-Talk

**DOI:** 10.3389/fpls.2012.00294

**Published:** 2012-12-31

**Authors:** Swetlana Friedel, Björn Usadel, Nicolaus von Wirén, Nese Sreenivasulu

**Affiliations:** ^1^Leibniz Institute of Plant Genetics and Crop Plant ResearchGatersleben, Germany; ^2^RWTH Aachen UniversityAachen, Germany; ^3^IBG-2: Plant Sciences, Institute of Bio- and Geosciences, Forschungszentrum JülichJülich, Germany

**Keywords:** abiotic stress, *Arabidopsis*, reverse engineering, systems biology, stress tolerance, yield

## Abstract

Understanding the global abiotic stress response is an important stepping stone for the development of universal stress tolerance in plants in the era of climate change. Although co-occurrence of several stress factors (abiotic and biotic) in nature is found to be frequent, current attempts are poor to understand the complex physiological processes impacting plant growth under combinatory factors. In this review article, we discuss the recent advances of reverse engineering approaches that led to seminal discoveries of key candidate regulatory genes involved in cross-talk of abiotic stress responses and summarized the available tools of reverse engineering and its relevant application. Among the universally induced regulators involved in various abiotic stress responses, we highlight the importance of (i) abscisic acid (ABA) and jasmonic acid (JA) hormonal cross-talks and (ii) the central role of *WRKY* transcription factors (TF), potentially mediating both abiotic and biotic stress responses. Such interactome networks help not only to derive hypotheses but also play a vital role in identifying key regulatory targets and interconnected hormonal responses. To explore the full potential of gene network inference in the area of abiotic stress tolerance, we need to validate hypotheses by implementing time-dependent gene expression data from genetically engineered plants with modulated expression of target genes. We further propose to combine information on gene-by-gene interactions with data from physical interaction platforms such as protein–protein or TF-gene networks.

## Introduction

During their growth and development plants are constantly exposed to various kinds of environmental stimuli and stresses due to their sessile lifestyle. Prominent abiotic stress factors affecting plant growth and yield relate to the shortage of water, the exposure to low or high temperatures, high salinity or nutrient deficiencies (Sreenivasulu et al., 2007; Lobell et al., 2011; Mir et al., 2012). Combinations of stresses such as drought and heat, as well as compounding effects of several abiotic and biotic stresses impact plant development more severely than single stress effects leading to severe yield losses (Atkinson and Urwin, 2012). Among the most prominent physiological alterations in response to abiotic stress is a substantial induction of the universal stress hormone abscisic acid (ABA; Sreenivasulu et al., 2012), which is also involved in the cross-talk between abiotic and biotic stresses (Chan, 2012). Most importantly, ABA leads to stomata closure to prevent water loss and thereby to a lower photosynthesis rate. To allow plants acclimating to stress, ABA further induces desiccation tolerance mechanisms, e.g., by inducing intrinsic proteins such as late embryogenesis abundant and heat shock proteins with chaperone activity, RNA chaperones, and the redox machinery or by reprograming metabolism for an enhanced synthesis of compatible solutes (Ma and Bohnert, 2008). Plants that successfully withstand stresses as part of a tolerance mechanism need to constantly monitor the external milieu through appropriate sensing mechanisms and need to redefine the appropriate cellular response for readjusting the metabolism to fine tune growth and development. Genotypes that fail to respond to stress with acclimation processes will not only suffer from impaired growth but undergo proteolysis, lipid peroxidation, and cellular disintegration due to the release of reactive oxygen species, finally leading to senescence and cell death (Chinnusamy et al., 2004; Sreenivasulu et al., 2007; Moreno-Risueno et al., 2010; Ma et al., 2012). Unraveling the mechanistic processes of coordinated whole-plant growth requires interlinking genotype × environment interactions with the dynamics of plant responses in systems biology approaches. To get comprehensive overview of gradual and complex changes in plant responses to altering environmental stimuli, stress responses need to be investigated not only from a temporal and spatial perspective but also at a cellular, organ, and whole-plant level.

Systems biology is an upcoming field in the area of plant biology aiming at integrating data from different high throughput omics platforms such as transcriptome, metabolome, proteome, and phenomics to understand the regulatory structure and organization of plant responses and their inherent components (Moreno-Risueno et al., 2010; Cramer et al., 2011). In this review, we briefly summarize the existing resources and advancement that has been made in the development of suitable software required to analyze gene regulatory networks and we exemplify this by a case study of plant responses to abiotic stress. Further, we focus on the strengths of reverse engineering strategies (Emmert-Streib et al., 2012), required as an essential part of systems biology to gain a more complete picture on regulatory and metabolic processes under multiple environmental perturbations. Based on transcriptional responses of different types of plant tissue to challenging conditions, the complex interplay and cross talk between different tissues is investigated to understand the dynamics of a tissue/organ/organism under different environmental challenges. The implementation of “reverse engineering strategies” not only deepens the holistic view of abiotic stress responses but eventually will make the way forward to identify key targets for developing stress-tolerant crops by new genetic engineering strategies.

## Conventional Transcriptomics Resources to Study Abiotic Stress Responses

On the basis of publicly available expression data (Cooper et al., 2003; Gomez-Porras et al., 2007; Ma et al., 2007, 2012; Mentzen and Wurtele, 2008; Weston et al., 2008; Zeller et al., 2009; Narsai et al., 2010; Worch et al., 2011), systematic studies have been undertaken to identify stress-specific and tissue/cell-specific stress responses. Despite this large amount of data, we still lack the understanding of common overlapping responses among various abiotic stress stimuli “as universally induced” factors and how these commonly induced regulators within the defined stress regulons influence plant growth and adaptation. Systems biology approaches may fill this gap and further define the role of “stress regulons” that act across different abiotic stress factors.

In the era of post-genomics we are continuously challenged with a massive flood of gene expression data generated by whole-genome transcriptome platforms. These platforms allow studies on profiling quantitative RNA abundance at the genome-wide gene content level for various stress responses. The available expression data are mostly deposited in online repositories such as GEO (Barrett et al., 2007), NASCArrays (Craigon et al., 2004), PLEXdb (Dash et al., 2012), and ArrayExpress (Parkinson et al., 2007; Kapushesky et al., 2012). In parallel, various online query-oriented tools have been developed such as Genevestigator (Zimmermann et al., 2004), *Arabidopsis* eFP browser (Winter et al., 2007), RiceArrayNet (Lee et al., 2009), or *Arabidopsis* and rice coexpression data mining tools (Manfield et al., 2006; Horan et al., 2008; Ficklin et al., 2010; Movahedi et al., 2011) to extract development- and stress-specific regulons by implementing global normalization and clustering algorithms (Sreenivasulu et al., 2010). Correlation networks derived from plant ontogeny have been extended for cross-species comparison (Mutwil et al., 2011). Recently, gene homologs exhibiting highest sequence identity and most reminiscent expression patterns under stress have been identified across several species (Patel et al., 2012). A comprehensive overview of coexpression tools and their relevance has been provided (Usadel et al., 2009). When handling large transcriptome data sets, the following challenges may arise: (i) if we select candidate genes based on log-fold changes and significance levels and then cluster these, we may overlook genes with functional relevance for the metabolic and regulatory pathways of interest. (ii) On the other hand, if we keep threshold levels low, it becomes a hard task to cluster relevant genes, to find representative patterns and to define stress regulons. In this case, a compromise may be achieved applying a “Between Group Analysis” (BGA; Culhane et al., 2002). This type of multivariate analysis can be used, if the effect of different treatments, for instance stress conditions on plant growth traits of wild type, mutant, and/or transgenic plants shall be compared simultaneously across different developmental-stages. The absence of constraints with regard to the number of samples compared to the number of genes makes it the method of choice for several kinds of analysis. BGA is based on a reduction of dimensions by identifying the factors contributing most to a large variance in principle component analysis (PCA). Thereby classifying the sample groups of tissue/developmental-stages/stress treatments, followed by a projection of the corresponding genes contributing most to the variance in individual sample groups as additional elements in the PCA, help to identify highly regulated genes. In the subsequent step, BGA provides scores allowing a graphical representation of results and defining a list of those genes which contribute most to each sample group mapped on the individual (first, second etc.) principal components. In case of noisy expression data or data coming from different experimental platforms where sophisticated statistical methods cannot be applied, an alternative method, called rank product analysis, promises to generate biological meaningful interpretations (Hong et al., 2006).

## Reverse Engineering Strategies to Deduce Gene Regulatory Networks

The rapid progress in sequence technologies and the generation of in-depth transcriptome data from different stress-treated plant genotypes, tissues, or cell types provide an ideal resource for reverse engineering approaches. Inferring gene regulatory networks aims at identifying possibly all molecular interactions among genes, which would be the primary goal of the reverse engineering concept. Gene regulatory networks are employed to elucidate the biological process underlying a certain stress response and the topology of interactions among the regulators being involved. Reverse engineering is still at its infancy but has greater potential to vastly expand in the field of plant abiotic stress tolerance. The statistical principles for different modeling approaches have recently been reviewed in great detail (Emmert-Streib et al., 2012). The most straight-forward approach is the implementation of correlation analysis based on Spearman or Pearson coefficients with appropriate thresholds. By coexpression analysis a lot of significant interactions can be obtained with high correlation coefficients, where a part of them share common genes: for instance, if there are strong correlations between gene 1 and gene 2 and between gene 1 and gene 3, then the correlation between gene 2 and gene 3 will be high by default (even if gene 2 and 3 are not correlated in nature). Thus the number of false positive interactions is high due to inferred edges, which depends on the previously set threshold for correlation coefficients. Such an indirect correlation result can be verified by calculating a partial correlation which answers the question whether gene 2 and gene 3 are still correlated, if gene 1 was constant (or completely removed from the picture). Nevertheless, suitable thresholds need to be selected, which may be difficult in the case of partial correlations as one needs to find a (high) threshold for validation of a correlation and another (low) threshold for disappearance of correlations after using partial correlations. However, physiology is usually more complex and instead of one gene mediating the correlation between two target genes, it may be a multi-layered, complex matrix of interactions, which requires higher-order partial correlation or estimation approaches (Opgen-Rhein and Strimmer, 2007). Another recent development has been the application of regression techniques such as LASSO modeling, which has allowed to uncover complementary candidate genes involved in potato responses to hypoxia (Licausi et al., 2011), or in the case of *Arabidopsis* seed development to identify genes involved in mucilage and pectin formation (Vasilevski et al., 2012). Furthermore, algorithms based on Bayesian Network, linear regression or multivariate auto-regressive models, or Hidden Markov Models have been implemented to decipher gene networks from differences in gene expression levels (Dojer et al., 2006). Bayesian network is a probabilistic graphical model, which can deal with noisy expression data, but its accuracy depends on the number of genes and samples. Because of its high computational complexity this methodology can be used only for a small number of genes. However, within the past few years the consideration of heuristics, such as clustering (Dimitrakopoulou et al., 2011) or the use of prior knowledge (Ong et al., 2002), has enabled constructing larger Bayesian networks. Particularly promising are approaches build on correlation, partial correlation, or similar approaches to efficiently reduce the search space (Lebre, 2009). Therefore, Dynamic Bayesian Network (DBN) is able to infer interactions from time-dependent gene expression data and consider regulatory feedback-loops to identify causal relationships from different stress scenarios. However, DBN still requires improvement with respect to handling computational complexity in larger gene sets (Faith et al., 2007; Cantone et al., 2009). Ordinary differential equations (ODEs) relate changes in transcript abundances to external stress stimuli or to the genetic constitution of a plant and directly yield a directed graph with a few nodes indicating interactions among genes. However, ODEs do not allow assigning any meaning to the estimated parameters (Huang et al., 2010). Due to their high computational complexity ODE-based approaches are time consuming. A very promising approach, which can deal with large-scale networks and allows reducing the number of false positives, is mutual information. Mutual information can be considered as a generalization of correlation analysis by dealing with non-linear, non-monotonic dependencies among genes. Therefore, mutual information in theory has a higher sensitivity when interactions within a huge number of genes are investigated (Steuer et al., 2002). To infer gene regulatory networks, the information flow between pairs of genes is mathematically transformed into a mutual information matrix. However, the problem of indirect influence between genes remains the same as detailed for correlation networks. It is therefore advisable to reduce the number of false positive interactions in a second step, using modern theoretical information methods, such as Maximum Relevance Minimum Redundancy, MRNET (Meyer et al., 2008), Algorithm for the Reconstruction of Accurate Cellular Networks, ARACNE (Zoppoli et al., 2010), Context Likelihood of Relatedness, CLR (Faith et al., 2007), C3NET (Altay and Emmert-Streib, 2010) and Directed Information, DTI (Kaleta et al., 2010). The implementation of most of those algorithms can be found in the *minet*-package of BioConductor/R Software (Meyer et al., 2008). According to Emmert-Streib et al. (2012), MRNET and C3NET outperform CLR, ARACNE, and relevance networks (RELNET), but definitely require more computational effort. Table 1 lists useful bioinformatics tools that are related to the analysis of gene regulatory networks. In Table 2, advantages and disadvantages for reverse engineering methods are discussed. The networks derived from reverse engineering algorithms can be well visualized using BioLayout Express 3D, Cytoscape, Osprey, Ondex, ProViz, Pajek, or Medusa (Pavlopoulos et al., 2008).

**Table 1:** **Bioinformatic tools that are related to the analysis of gene regulatory networks**.

Name	Mathematical model	Description	Reference	
**(A) REVERSE ENGINEERING TOOLS**				
WGCNA	Weighted correlation	The R software package WGCNA is a comprehensive collection of R functions for performing various aspects of weighted correlation network analysis.	Langfelder and Horvath (2008)	
qp-Graph	Partial correlations	q-Order partial correlation graphs, or qp-graphs is useful for learning undirected graphical Gaussian Markov models from data sets in which the number of random variables *p* exceeds the available sample size *n*. In the case of microarray data it can be employed to reverse engineer a molecular regulatory network		
GeneReg	Linear model fitting	GeneReg is used to reconstruct time-course gene regulatory network. R package GeneReg reconstructs a gene regulatory network from short time-course gene expression data. A suitable application is the study of time-dependent biological processes such as cell cycle, cell differentiation, or causal inference	Huang et al. (2010)	
BoolNet	Boolean Networks	BoolNet or Boolean networks inference R package developed methods for synchronous, asynchronous, and probabilistic Boolean Network. It can be applied for reconstructing networks from time series and can be used for robustness analysis via perturbation (environmental or genetic) and identification and visualization of attractors in networks	Mussel et al. (2010)	
BNArray	Bayesian Network	BNArray: An R package for constructing gene regulatory networks from microarray data by using Bayesian network modeling.	Chen et al. (2006)	
GRENITS	Dynamic Bayesian network	The package GRENITS (Gene Regulatory Network Inference Using Time Series) offers four network inference statistical models using Dynamic Bayesian Networks and Gibbs Variable Selection: a linear interaction model, two linear interaction models with added experimental noise for the case where replicates are available and a non-linear interaction model	Morrissey et al. (2010)	
Minet	Mutual information	The R package *minet* provides a set of functions to infer mutual information networks from a microarray dataset. Four different entropy estimators are available in the package: empirical, Miller–Madow, Schurmann–Grassberger and shrink-, as well as four different inference methods, namely relevance networks, ARACNE, CLR, and MRNET. Also, the package integrates accuracy assessment tools, like F-scores, PR-curves and ROC-curves in order to compare the inferred network with a reference set.	(Meyer et al., 2008)	
Parmigene	Mutual information	The R package parmigene (PARallel Mutual Information estimation for GEne NEtwork reconstruction) implements a mutual information estimator based on k-nearest neighbor distances that is minimally biased with respect to the other methods and uses a parallel computing paradigm to reconstruct gene regulatory networks. parmigene gives more precise results than existing softwares at strikingly lower computational costs.	(Sales and Romualdi, 2011)	
DTI	Mutual information	An R package including functions for inference of gene regulatory networks from microarray data. Directed information approach enables to reconstruct a directed graph.	(Kaleta et al., 2010)	
C3NET	Mutual information	R package Conservative cause core (C3NET) algorithm is based on mutual information and composed of two steps. The first step confers an elimination of non-significant edges, in the second step genes are only connected if their shared significant mutual information value is at least for one of these two genes maximal with respect to all other genes	Altay and Emmert-Streib (2010)	
Name	Kind of interactions network	Description	Taxon	Reference
**(B) NETWORK EXPLORATION TOOLS FOR *ARABIDOPSIS* AND OTHER CROP PLANTS**				
CORNET	TF-to-gene	The TF tool retrieves regulatory interactions from AGRIS and from CORNET microarray data. The resulting network is represented in Cytoscape with possibilities to see localization, TAIR functional descriptions, Gene Ontology, Plant Ontology, MapMan pathways and processes, protein domains, PubMed IDs and phenotypes. Link out to other external databases by right-clicking the nodes in Cytoscape	*A. thaliana*, *Z. mays*	De Bodt et al. (2012)
AtRegNet	TF-to-gene	AtRegNet tool of AGRIS: *Arabidopsis* Gene Regulatory Information Server contains 11,355 direct interactions between known transcription factors and target genes in *A. thaliana*. It is based on TAIR9 annotations	*A. thaliana*	Yilmaz et al. (2011)
CORNET	Protein–protein interaction	PPI tool interrogates available protein–protein interaction databases (both experimental and predicted interactions) and the AraNet probabilistic functional gene network. It includes, e.g., ArathReactome, AtPID, MINT, Yeast-2-hybrid Interactome, and so on. The resulting network is represented in Cytoscape with possibilities to see localization, TAIR functional descriptions, Gene Ontology, Plant Ontology, MapMan pathways and processes, protein domains, PubMed IDs, and phenotypes. Link out to other external databases by right-clicking the nodes in Cytoscape	*A. thaliana*, *Z. mays*	De Bodt et al. (2012)
PMN	Metabolic network	The Plant Metabolic Network (PMN) provides a broad network of plant metabolic pathway databases that contain curated information from the literature and computational analyses about the genes, enzymes, compounds, reactions, and pathways involved in primary and secondary metabolism in plants	Over 350 plant species	Mueller et al. (2011)
MetNet	Metabolic network, TF-to-gene, evidence network	The MetNet database (MetNetDB) contains integrative information on networks of metabolic and regulatory interactions. Types of interactions in MetNetDB include transcription, translation, protein modification, assembly, allosteric regulation, translocation from one subcellular compartment to another. Other fields describing the interactions are subcellular localization, confidence, directionality, references, evidence, and synonyms. Data on entities (DNA, RNA, polypeptides, protein complexes, metabolites) are derived from web databases (TAIR, GO, MapMan/GabiPD, PPDB, AMPDB, AtNoPDB, AraPerox, PLprot, BRENDA, ChEBI, PubChem, KEGG, NCI, NIST MS library), in some cases with additional annotation by experts	*A. thaliana* and *G. max*	Sucaet and Wurtele (2010)
AtCOECis	TF-to-gene	Transcriptional regulatory network is reconstructed here based on cis-regulatory elements and coexpression network	*A. thaliana*	Vandepoele et al. (2009)
ATTED-II	Coexpression	ATTED-II builds coexpression network allows searching coexpression network for individual target genes of interest or co-expressed network for each functional bin. Also abiotic, biotic, hormone treated gene expression data deposited for deriving coexpression network	*A. thaliana*	Obayashi and Kinoshita (2010), Obayashi et al. (2011)
Genevestigator	Coexpression	Pearson correlation coefficient is taken as the measure of similarity between genes, both for identifying co-expressed genes as well as to define the pairwise correlation between genes in the plot. This score is calculated based on log2-scaled expression data that is processed from the Genevestigator database	*A. thaliana, H. vulgare, N. tabacum, O. sativa, T. aestivum, Z. mays*	Zimmermann et al. (2004)
MapMan	Visualization	MapMan brings its own hand-curated ontology which helps in exploring and understanding plant networks. The ontology aims to be redundancy-free aiding in simple graphical network visualization.	*A. thaliana*, *Z. mays H. vulgare*	Lohse et al. (2010)
Planet	Coexpression	Using multiple different plant species and mutual best ranked coexpression as well as domain information and MapMan terms it is possible to find conserved correlations between plants	*A. thaliana, G. max, M. sativus, Poplar, O.sativa, H. vulgare, T. aestivum*	Mutwil et al. (2011)
Corto	Coexpression	Corto is a network building and visualization tool which comes pre-loaded with several plant specific array sets. Its prime role is network reconstruction of user supplied data sets however. CorTo applies simple correlation, partial correlation, lasso regression, and mutual information. A query centered target gene network can be visualized and explored. All nodes can be color coded based on MapMan categories	Any species where high throughput data is available	Giori unpublished. Available from usadellab.org
CORNET	Coexpression	Using one or more precompiled expression datasets the correlation between gene expression profiles will be calculated. There are possibilities to identify threshold values for acceptance of interactions. The result can be visualized as a graph in Cytoscape providing all possible gene annotations	*A. thaliana*, *Z. mays*	De Bodt et al. (2012)
AraNet	Evidence network	AraNet yields all neighbors to query genes, based on coherence of query genes, which is measured by the area under the ROC curve AUC from 0.5 to 1. Top neighbors would be good candidates for your follow-up screen, network-guided focused screen. As evidence, AraNet uses a probabilistic functional gene network of *Arabidopsis* thaliana, constructed by a modified Bayesian integration of 24 types of “omics” data from multiple organisms, with each data type weighted according to how well it links genes that are known to function together in *Arabidopsis* thaliana. Each interaction in AraNet has associated LLS that measures the probability of an interaction representing a true functional linkage between two genes	*A. thaliana*	Lee et al. (2010)
STRING	Evidence network	STRING is a database of known and predicted protein–protein interactions. The interactions include direct (physical) and indirect (functional) associations; they are derived from genomic context, conserved coexpression, HT experiments, and text mining	1133 organism including plants *A. thaliana*, *O.sativa* etc.	Szklarczyk et al. (2011)
EVEX	Biomolecular interaction based on textmining	EVEX or Event Extraction is a text mining resource built on top of PubMed abstracts and PubMed Central full texts. It contains over 34 million biomolecular events among more than 67 million automatically extracted gene/protein name mentions. The text mining data has been enriched with gene normalization results, covering more than 42% of all gene/protein names. EVEX presents both direct and indirect associations between genes and proteins, enabling explorative browsing of relevant literature	Broad range of species, including plant species	Van Landeghem et al. (2012)
MetaCrop	Metabolic network	MetaCrop is a manually curated repository of high-quality data about plant metabolism, providing different levels of details from overview maps of primary metabolism to kinetic data of enzymes. It can be accessed via web, web services and an add-on to the Vanted software	It contains seven major crop plants and two model plants	Schreiber et al. (2012)

**Table 2:** **Advantages and disadvantages of reverse engineering methods**.

Model	Advantages	Disadvantages
Boolean network	Large-scale network Better handling of computational complexity	Deterministic description Binary abstraction with information loss
Bayesian network	Handle incomplete and noisy data Learning about causality Integrating of prior knowledge Interpretation of network topology (hubs, modules)	Computational complexity Handling of feedback-loops not possible
Dynamic Bayesian network	Handling of feedback-loops and incomplete and noisy data Learning about causality Integrating of prior knowledge Handling of time series and causal relationship from perturbations Interpretation of network topology (hubs, modules)	Computational complexity Deriving regulatory networks using a multivariate approach considering only the best-scoring network due to limitation of computational time
Differential equations	Handling of negative feedback-loops Great physical accuracy Good performance	Computational complexity Small number of genes Require experimental parameters
Correlation analysis	Large-scale network Interpretation of network topology (hubs, modules)	Dependency of accuracy on the set of thresholds Integration of prior knowledge For linear or monotonical interactions
Mutual Information	Large-scale network Better handling of computational complexity through pairwise comparison Identifying causal relationship of TF-gene prediction Handling of feedback-loopsHandling of feedback-loopsHandling of feedback-loopsHandling of feedback-loops Reducing false positives and extract causal rather than associative links in gene networks Non-linear and non-monotonically dependencies	Dependency of accuracy on the set of thresholds Integration of prior knowledge

**Figure 1:**
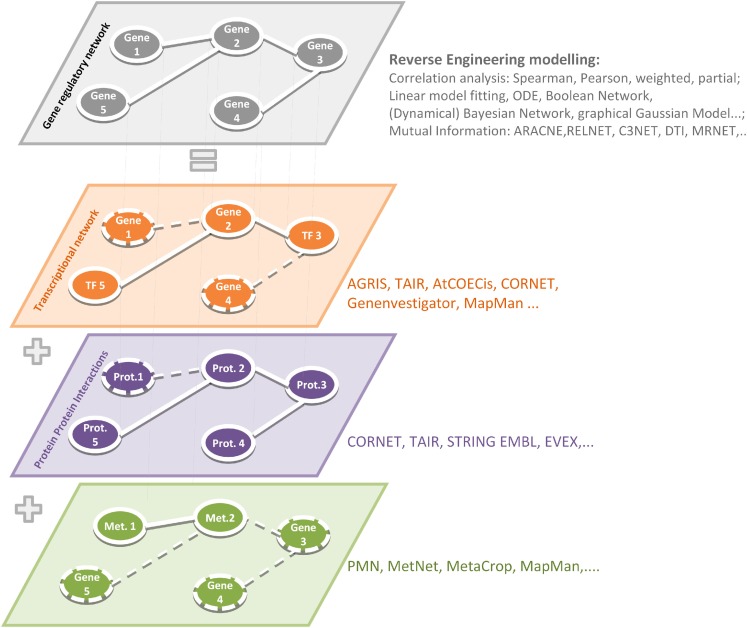
**Reverse engineering strategies to simulate gene regulatory networks and reconstructed relationship at other physical interactions such as TF-to-gene, protein–protein, and metabolic networks**. The application of relevant softwares and algorithms has been summarized in Table 1.

Depending on the chosen experimental conditions (tissue-, condition-, treatment-, development-dependent studies) and data sets (Chip-Seq, RNA-Seq, microarrays) different biological interpretations can be made based on gene-by-gene networks, for the interpretation of which a profound biological knowledge plays the most important role. Before interpreting gene-by-gene networks, it is important to refer a gene regulatory network to other available data, e.g., on physical TF-to-gene or protein–protein interactions. Reconstructed relationships between genes can be caused by transcriptional and posttranscriptional modifications (TF-to-gene) or can be explained by the participation of one gene product in different protein complexes (because of protein–protein interactions) or in several metabolic pathways (Metabolic Networks), refer Figure 1.

As a part of systems biology approaches, several methods have been developed to construct genome-wide gene networks using heuristic cluster chiseling algorithms and correlation coefficients. These networks have been derived from whole-genome coexpression networks in *Arabidopsis* and six other important crop plants (Lee et al., 2009; Mutwil et al., 2011), which allows cross-species comparisons based on time series networks. Gene networks in *Arabidopsis* (curated at AtGenExpress) derived from genome-wide gene expression data using the Affymetrix platform as well tiling arrays designed for the assessment of plant responses to environmental perturbations, help to understand the fundamental stochastic processes underlying individual cell and tissue type-specific responses to various abiotic stress factors (Kilian et al., 2007; Zeller et al., 2009). Since transcriptional networks already vary during plant development, stress-induced transcriptional networks must be supposed to vary dynamically depending on the plant developmental stage at which the stress is experienced. Hence, transcriptional networks can not only explain the phenotypic plasticity of plant adaptation to different abiotic stresses but also indicate core regulatory mechanisms of TF networks which are either shared across species or unique to species or even genotypes.

## Multi-Layered Regulation in Reverse Engineering

Gene-by-gene networks can be modeled and simulated at qualitative and quantitative levels to unravel abiotic stress responses. The data may come from any type of genome-wide expression analysis, such as DNA microarrays, protein expression, miRNAs expression, ChIP-chip, ChIP-Seq data, or RNA-Seq data. As outlined above, the systematic analysis of large-scale gene expression data from the model plant *Arabidopsis* provides a rational way to increase the knowledge of stress perception and stress tolerance by identifying the key target genes. At first, the gene regulation at transcriptional level could be associated with transcription factor (TF)-target gene relationships by constructing gene regulatory networks via reverse engineering methodologies that integrate *in vivo* confirmation of new regulatory interactions using ChIP-chip (Faith et al., 2007; Wu and Chan, 2012). This approach can be further extended to other levels of gene regulation and thereby consider the role of small RNAs (miRNA, siRNA, and long non-coding RNAs) or epigenetic-based chromatin modifications, so that such an extended analysis of abiotic stress responses would provide a holistic view of regulatory events. TILING array-based transcriptome data provide important insights not only into coding ORFs but also into the activation of pseudogenes and retro-transposons under various abiotic stresses. In the case of ABA treatments, this analysis suggests the importance of additional levels of regulation conferred by non-coding RNA and epigenetic regulation (Zeller et al., 2009). TILING arrays (Agilent 60mer probe) and AGRONOMICS1 (a new Affymetrix *Arabidopsis* array with 25 and 35mer probes) can also be used for interdisciplinary approaches such as chromatin immunoprecipitation on chip (ChIP-on-chip) to identify binding sites of TF (Rehrauer et al., 2010; Sreenivasulu et al., 2010). TILING arrays are also useful if an altered DNA methylation status of interesting loci corresponds to changes in steady-state mRNA levels (Aceituno et al., 2008; Chinnusamy et al., 2008; Chinnusamy and Zhu, 2009). In this regard, conducting genome-wide epigenome studies to scan for cytosine-phosphate-guanine (*CpG*) islands under various stress treatments will be an important future topic. Besides, microarray platforms can be used to identify stress-regulated microRNAs, small RNAs, and long non-coding RNAs (Sunkar et al., 2007). All in all, these combinatorial technologies eventually allow a global understanding of the complex regulatory networks (regulatory role of hormones/TFs – small RNAs – epigenetics) to generate a specific abiotic stress response. Such case studies are not yet available in the area of stress genomics and therefore we focussed here on gene regulatory networks.

## Unraveling Transcriptome Dynamics of Abiotic Stress Responses in *Arabidopsis* by Reverse Engineering: A Case Study

**Figure 2:**
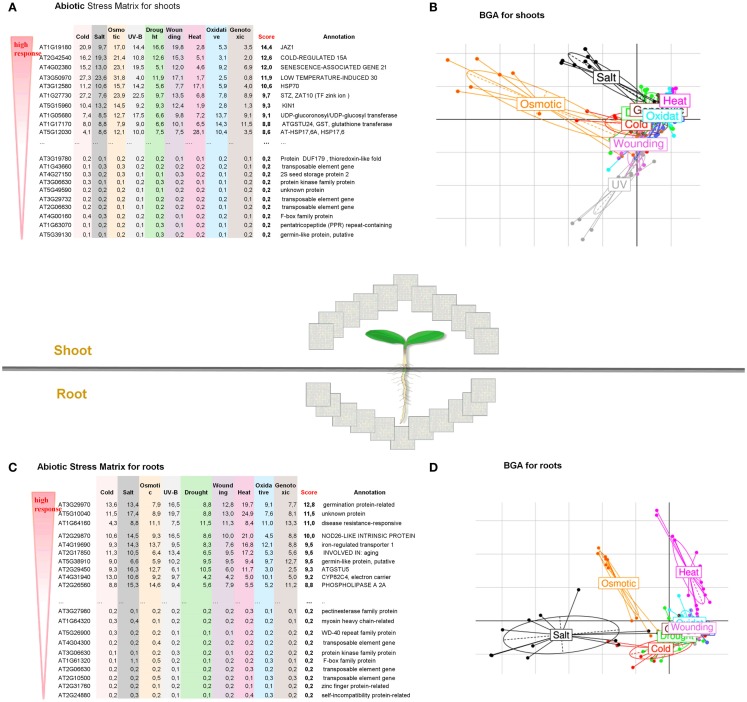
**Global overview of abiotic stress responses in roots and shoots of *A. thaliana***. Abiotic Stress Matrix for shoot **(A)** and root tissues **(C)**. Values are calculated as a sum of absolute log-fold changes over all time points. The score is calculated as median over all abiotic stress conditions. Between Group Analysis for the whole transcriptome of shoot **(B)** and root **(D)**. The larger distance between stress and control, the stronger the transcriptome reprograming under a given stress. Higher variance for shoots is noted for Osmotic > Salt > UV > Wounding. Higher variance for roots is noted for Salt > Osmotic > Heat.

In the present case study, we performed a large-scale analysis of changes in the transcriptome of *Arabidopsis thaliana* when subjected to abiotic stress (i) to find general patterns in gene expression data typical for roots and shoots, (ii) to identify genes that are highly inducible and represent a common response to different abiotic stress conditions, and (iii) to reconstruct gene regulatory networks for the top regulated gene sets in root and shoot tissues. We analyzed 272 microarray samples of *A. thaliana* for shoots and roots obtained from nine different abiotic stress conditions analyzed at six time points (0.5–24 h). The data sets are publicly available at GEO [AtGenExpress abiotic stress series, under GEO NCBI Acc. Numbers GSE5628 (heat stress), GSE5627 (wound stress), GSE5626 (UV-B stress), GSE5625 (genotoxic stress), GSE5624 (drought stress), GSE5623 (salt stress), GSE5622 (osmotic stress), GSE5621 (cold stress), and GSE5620 (respective control tissues; Kilian et al., 2007)]. In order to find stress-induced regulons, a BGA was performed, which highlighted that the first two principal components projected almost 60% of all variance in gene expression, indicating dramatic transcriptional reprograming events during the exposure of *Arabidopsis* plants to different kinds of abiotic stress (Figure 2). Among the various abiotic stress responses, osmotic stress and salt stress caused the strongest reaction in both leaf and root tissues. Interestingly, heat treatment also caused a drastic transcriptional reprograming in roots, while almost no effect was seen after UV treatment (Figures 2B,D). These stress-specific alterations in the transcriptome of above-ground and below-ground plant tissues are quite diverging. Furthermore, these BGA plots also indicated stress responses that overlap between drought, oxidative, cold, genotoxic, and wounding treatments and may therefore share common underlying regulators (Table S1 in Supplementary Material). To identify the most responsible genes in every abiotic stress treatment, we calculated a new *Abiotic Stress Matrix* to score all genes according to their global impact on the response to different stresses. For every gene a score has been calculated according to the following formula:

$$S^{\text{stress}}=\sum_{t\in \left\{0,5,1,3,6,12,24h\right\}}\log_{2}\left|\frac{{\text{stress}}_{\text{t}}}{{\text{control}}_{\text{t}}}\right|$$

where stress*_t_* and control*_t_* are normalized expression values for nine selected abiotic stress treatments and the respective control. The resulting stress matrix encompasses all genes present on the Affymetrix ATH1 array and assayed under nine stress conditions in roots and shoots and identified the top100 regulated and bottom100 unregulated gene sets (Table S1 in Supplementary Material). We first took the median of the expression value over all conditions (0.5–24 h), calculated fold changes (based on median expression in stress versus control) and then deduced ranks to order the gene sets along their impact on the stress response. This resulted in a group of top ubiquitous abiotic stress-regulated genes (Figures 2A,C and Table S1 in Supplementary Material).

**Figure 3:**
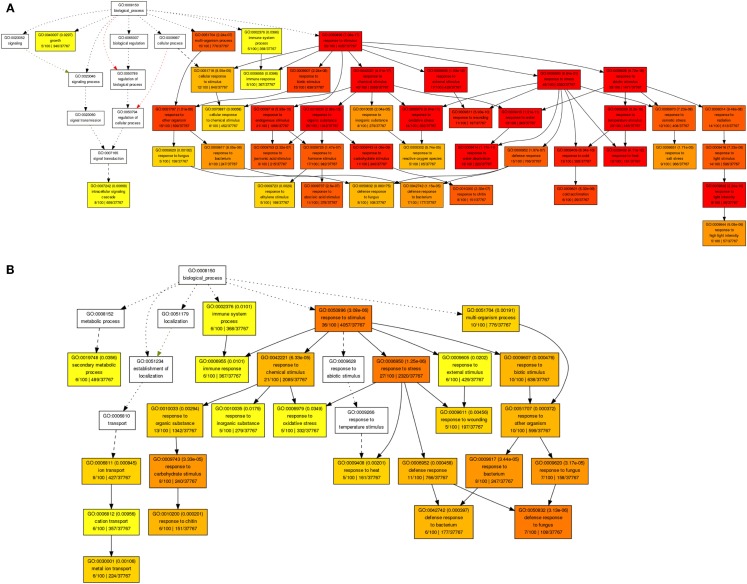
**Gene ontology enrichment of top 100 abiotic stress-regulated genes in shoots (A) and roots (B) using AgriGO database**.

By revisiting this comprehensive transcriptome data set of short-term shoot abiotic stress responses using Gene Ontology (GO) analysis we identified an enrichment of genes involved in plant responses to reactive oxygen species, water deprivation, heat, light, or cold, as well to carbohydrate limitation or biotic stimuli (Figure 3A). Within the root tissue, responses to heat, oxidative stress, carbohydrate limitation, and biotic stimuli were found to be characterized by ubiquitous abiotic stress-regulated gene sets (Figure 3B). Among the most stress-responsive gene sets, transcripts involved in protein degradation, or coding for AAA-type, cysteine proteases, serine proteases, or selected members of RING-domain E3 ubiquitin ligases were found to be highly induced in both root and shoot tissues. Another large class of proteases that belongs to the E3.SCF F-box family was found to be non-responsive to many of these abiotic stress treatments. Thus, irrespective of stress-specific stimuli, there seems to be a conserved abiotic stress-responsive cascade triggering cell death under unfavorable growth conditions, possibly due to the release of reactive oxygen species and damage of membrane lipids, leading to cellular disintegrity, protein degradation, and apoptosis (Ma and Bohnert, 2008). Consequently, a transgenic strategy should consider secondary effects by creating an effective scavenging system at the intracellular compartment level (cytosol, mitochondria, and plastidial) to quench released reactive oxygen species generated during heat, drought, or a combination of these stresses (Mittler, 2006) to finely modulate PCD pathways.

**Figure 4:**
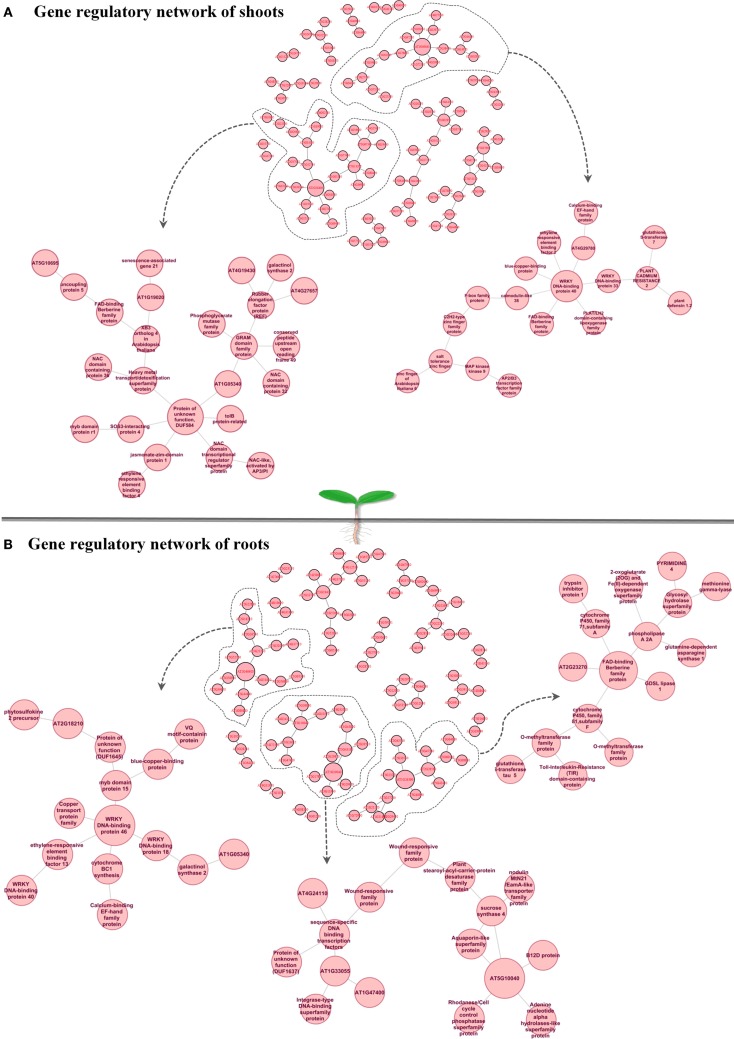
**Gene regulatory network of shoots (A) and roots (B) highlights “cross-talk” between the top 100 stress-inducible genes which have been defined from Abiotic Stress Matrix**. Within the shoot gene regulatory network two hub genes AT2G28400 (unknown protein) and AT1G80840 (WRKY40) and their connected sub-networks are shown in detail. Within the root gene regulatory network three hub genes (1) AT2G46400: ATWRKY46, (2) AT5G10040 (unknown protein), (3) AT1G26380 FAD-binding domain-containing protein and their connected sub-networks are shown in detail.

Understanding dynamic alterations in the cross talk between phytohormones and TFs in response to different abiotic stress factors and their importance for optimum plant growth is a major challenge. In this respect, systems biology approaches will remain pivotal. With regard to hormonal regulation, we identified in shoots an enrichment of gene expression changes in the stress-related phytohormones ABA, jasmonic acid (JA), and ethylene (Figure 3A). In roots, we found JA, ethylene, and various abiotic stress-responsive WRKY TFs (as classified by MapMan bins) to be enriched. By combining a weighted gene coexpression network of the abiotic stress transcriptome, ABA, JA, and ethylene signaling hubs were identified to be of central importance for the integration of different abiotic stress stimuli (Cramer et al., 2011). However, in the last two decades ABA has been described as an important phytohormone signaling in abiotic stress responses (esp. drought, osmotic, and low temperature) while JA, ethylene, and salicylic acid were considered to play central roles in biotic stress responses (Lorenzo et al., 2003; Robert-Seilaniantz et al., 2011; Sreenivasulu et al., 2012). The recently increasing knowledge emerging from genomic studies clearly points out that ABA, JA, and ethylene are involved in the cross-talk of abiotic and biotic stress responses. Thus, unraveling converging points and key regulators involved in the cross-talk between these hormones and signaling pathways under both abiotic and biotic stress responses will help to understand how ethylene, JA, and ABA operate synergistically in mediating general stress response. Moreover, the role of these signaling molecules need to be investigated under short-term versus long-term stress responses to understand the regulation of subsets of cascades involved in defense responses and growth acclimation under stress. Reverse engineering approaches will not only help to unravel which key regulators determine the expression of subsets of genes responding to certain environmental perturbations, but also allow identifying key targets causing a perturbation in plant growth. Such targets may eventually be used in the generation of stress-tolerant plants to promote sustainable growth and stable yield under various stress conditions. Subsequently, the network inference could be employed to confirm the major targets in the genetic reprograming required to achieve sustainable growth under challenging conditions. To investigate the cross-talk between the most stress-inducible genes in *Arabidopsis* shoots and roots, we reconstructed a gene regulatory network (Figures 4A,B), using the MRNET algorithm.

In shoots of stress-exposed *Arabidopsis* plants the genes AT1G80840 and AT2G28400 were identified as central hub genes (Figure 4A), potentially involved in coordinating the overall abiotic stress response. Their gene regulatory network highlights a “cross-talk” between the top 100 stress-inducible genes which have been defined from the Abiotic Stress Matrix. Two parts of the network with the hub genes AT2G28400 (unknown protein DUF584) and AT1G80840 (*WRKY40* pathogen-induced TF) are shown in detail. In the ontology “Molecular Function,” both networks are characterized by the overrepresented category GO:0003700 – TF activity (FDR < 0.05, in both cases). Considering the “Biological Function” ontology AT2G28400 network shows an enrichment of GO:0042221 – response to chemical stimulus (FDR < 0.01) and the *WRKY40* subnetwork shows an enrichment of GO:0009611 (FDR < 0.01) – response to wounding. WRKY40 is known as a pathogen-induced TF involved in repressing basal defense responses to *Pseudomonas syringae* with a conserved function in both, dicots and monocots (Shen et al., 2007). WRKY40 forms homomeric protein complexes and binds *in vitro* to W-boxes in promoter sequences. However, its role in abiotic stress responses has not been analyzed so far. WRKY40 is a central negative regulator, inhibiting the expression of ABA-responsive genes, such as *ABI5*, *ABI4*, *ABF4*, and thereby repressing a part of the ABA signaling pathway (Shang et al., 2010). Recent data suggest that WRKY40 forms a highly interacting regulatory network with WRKY18 as well with WRKY60 and modulates gene expression in plant defense and abiotic stress responses (Chen et al., 2010). WRKY40 itself is known to be modulated by the phytohormones ABA and JA (Galletti et al., 2008; Chen et al., 2010). In our reverse engineering approach, we identified that WRKY40 is a central node in the regulation of abiotic stress responses in shoots and forms a gene regulatory network with other TFs such as ERF2 (a positive regulator of JA-regulated defense genes) and WRKY33 (involved in abiotic stress responses with high sensitivity to ABA) as well as other gene sets which include lipoxygenase 4, calmodulin-like 38, tripeptidyl peptidase 2, and a blue copper-binding protein (Figure 4A). All promoters of these target genes carry the known W-box cis-element TTGAT/C. A second central hub of abiotic stress responses in shoots is AT2G28400 (T1B3.8), which encodes an unknown protein named DUF584. From our analyses it interacts with the unknown protein AT1G05340, a heavy metal transport/detoxification superfamily protein, the tolB protein-related and SOS3-interacting protein 4, RD26 and JAZ1 (having the highest score in the Abiotic Stress Matrix in shoots). The interaction of AT2G28400 with RD26 has been previously confirmed in a *RD26* overexpression study (Fujita et al., 2004). The receptor for jasmonates was found to be CORONATINE INSENSITIVE1 (COI1), which is a F-box protein essential for jasmonate responses. COI1 interacts with multiple proteins to form the SCF-COI1 E3 ubiquitin ligase complex (Yan et al., 2009). JA-Ile forms a ternary complex with SCF-COI1 and JASMONATE ZIM-DOMAIN (JAZ) proteins, which are repressors of the TF MYC2, consigning the JAZ proteins to 26S proteasome-mediated degradation, hence facilitating stress-inducible gene expression through MYC2 (Fonseca et al., 2009; Wasternack and Kombrink, 2010; Wasternack and Xie, 2010). JAZ proteins are repressors of JA-mediated nicotine biosynthesis, involved in defense versus growth responses with a clear antagonistic regulation by JA and GA (Kazan and Manners, 2012). Taken together, these results suggest that many of these key regulators modulate growth by interfering in the trade-off between abiotic and biotic stress responses.

**Figure 5:**
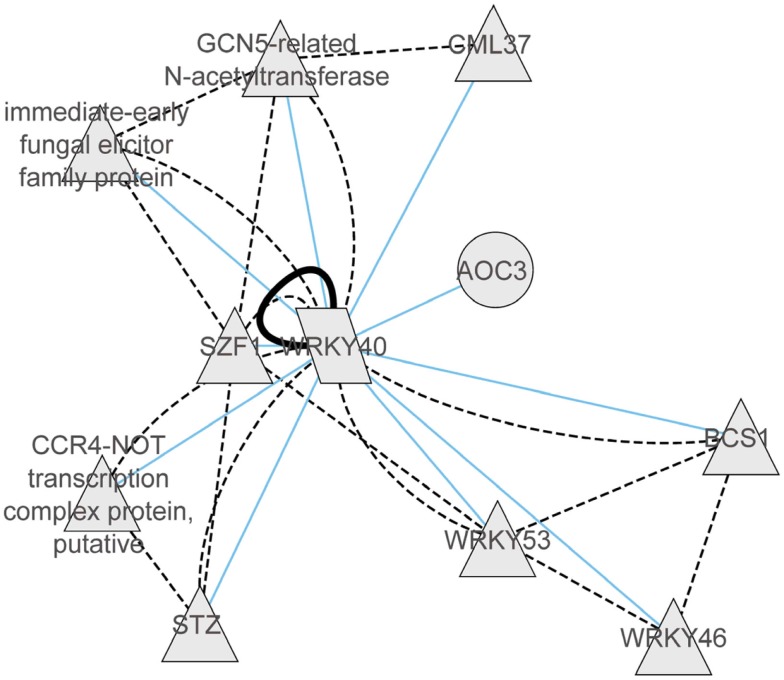
**Coexpression gene network and protein–protein interaction network of WRKY40 derived from CORNET database (De Bodt et al., 2012)**. Within the coexpression network blue lines represent correlations of >0.9. The protein–protein interaction network is represented by black solid lines (validated protein interactions) or by dotted black lines (predicted protein–protein interactions).

With regard to the regulatory networks in stressed *Arabidopsis* roots, AT2G46400 (AtWRKY46), AT1G26380 (FAD-binding domain-containing protein), and AT5G10040 (unknown protein) were identified as central hubs, with a high probability to coordinate stress responses (Figure 4B). In the category “Molecular Function” the AtWRKY46 network is characterized by the enrichment term GO:0003700 – transcriptional factor activity (FDR < 0.01) and GO:0009743 – response to carbohydrate stimulus; the AT1G26380 network is enriched by the GO:0003824 catalytic activity, GO:0009697 response to biotic stimulus, and GO:0051707 response to other organisms. There are no significant GO terms for the AT5G10040 network. Interestingly, similar to shoots, WRKY46 regulates another member of the WRKY family, namely WRKY18, and a member of the ETHYLENE RESPONSIVE FACTORS (ERF), ERF13. Other strongly interacting partners of WRKY46 are the TF MYB15 and the copper transport protein BCS1. In general, there was only 10% of overlap between roots and shoots in the response to abiotic stress. In both tissues, however, the central hub networks are represented by WRKY-homologs WRKY40 in shoots and WRKY46 in roots. Nevertheless, both WRKYs appear to regulate, in turn, TFs of the ERF and WRKY families, suggesting a partial overlap in their regulation of downstream targets. Based on the predicted protein–protein interaction and coexpression networks from the CORNET database, we identified that WRKY40 acts as a central regulator and forms complexes with WRKY46, WRKY53, SZF1, STZ, CCR4, GCN5, GML37, and an immediate-early fungal elicitor family protein (Figure 5).

**Figure 6:**
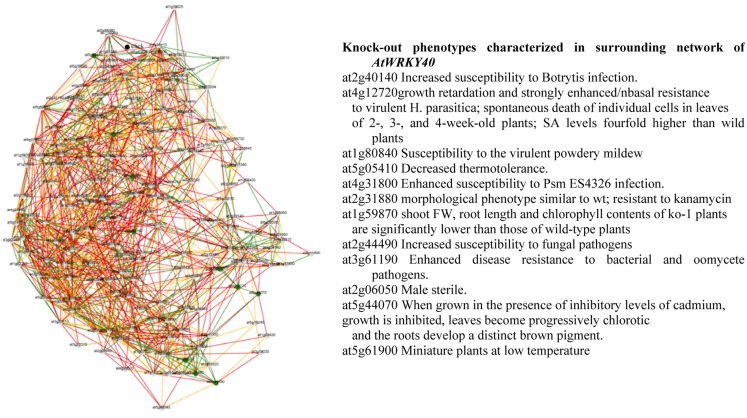
**Gene coexpression network and knock-out phenotypes characterized in surrounding network of AtWRKY40 derived from PLANET database (Mutwil et al., 2011)**.

From the Planet database, information can be obtained on knock-out phenotypes of genes involved in the regulatory network of WRKY40 and WRKY46. Interestingly, the phenotypes of the target genes of both WRKYs were predicted to show increased susceptibility to pathogens, decreased thermo tolerance, chlorotic leaves, and inhibited growth (Figure 6). These results suggest that both, abiotic and biotic stress factors share at least in part common pathways of the general stress-responsive machinery. It remains interesting to see how this general stress-regulatory machinery will be connected to individual stress-specific pathways and thus regulating key traits controlling plant growth and development under stress. Conclusions and hypotheses generated from such reverse engineering strategies need to be validated by (i) identifying the respective mutants and/or fine-tuned manipulation of key regulatory targets in transgenic approaches and (ii) eventually employing these mutants or transgenics in systems biology approaches to validate the selected regulatory cascades.

## Future Perspectives

Exploring gene regulatory networks derived from genome-wide data sets for various stress responses is an important milestone in the era of green biotechnology. Novel regulatory components within these networks can be identified as central hubs or as key candidate genes to further explore their functional relevance using systems biology approaches. Undoubtedly, the analysis of integrated genome-wide networks has helped immensely to identify key target genes for crop improvement under ambient conditions (Ferrier et al., 2011), but it has even greater potential in the area of defense responses to abiotic stresses and for identifying key targets promoting growth stability. However, extending such knowledge to finally predict plant growth responses to stress requires an even more comprehensive understanding of underlying regulatory networks, which can only be done by integrating analyses on phytohormones to elucidate hormone-TF networks from the cell type to the whole-plant level. This may be achieved either by direct measurements of relevant phytohormone species, which is a challenge regarding the large number of more and less physiologically active forms of phytohormones or by employing reporter genes, which sometimes allow a cell type-specific resolution of hormonal gradients but may be confounded by the interaction with other hormones or by variable hormone sensitivities of stressed tissues. To overcome the gap between the identification of stress-regulated genes or hubs and the manipulation of yield-determining genes for improved crop growth under challenging environmental conditions, we need further knowledge on (a) how stress cues become active and integrated in plant developmental programs at different growth stages and (b) which are the specific and which are the common regulatory nodes involved in promoting growth under multiple abiotic and biotic stress factors. Such a systematic and integrative knowledge will be required to engineer and develop multi-stress-tolerant plants. For instance, from the above-mentioned data-mining approaches we start realizing the importance of ABA and JA interactions in several abiotic stress treatments. This is not achieved when looking at the individual role of these hormones, as presently done when dissecting the role of ABA in abiotic and of JA for biotic stress responses. Thus, combinatory stress treatments are required to better discriminate specific from ubiquitous stress response pathways. Discoveries obtained by reverse engineering approaches may then allow us to forge ahead toward the generation of a new generation of stress-tolerant plants.

## Conflict of Interest Statement

The authors declare that the research was conducted in the absence of any commercial or financial relationships that could be construed as a potential conflict of interest.

## Supplementary Material

The Supplementary Material for this article can be found online at http://www.frontiersin.org/Plant_Systems_Biology/10.3389/fpls.2012.00294/abstract

Supplementary Table S1 **List of top ranked abiotic stress-induced gene matrix and central hubs of abiotic stress response in shoots and roots of *A. thaliana***.
